# Plasma and Milk Pharmacokinetics and Estimated Milk Withdrawal Time of Tolfenamic Acid in Lactating Sheep

**DOI:** 10.1002/vms3.70047

**Published:** 2024-09-25

**Authors:** Orhan Corum, Kamil Uney, Devran Coskun, Duygu Durna Corum, Gul Cetin, Muammer Elmas

**Affiliations:** ^1^ Department of Pharmacology and Toxicology Faculty of Veterinary Medicine University of Hatay Mustafa Kemal Antakya Hatay Türkiye; ^2^ Department of Pharmacology and Toxicology Faculty of Veterinary Medicine University of Selcuk Konya Türkiye; ^3^ Department of Pharmacology and Toxicology Faculty of Veterinary Medicine University of Siirt Siirt Türkiye; ^4^ Department of Pharmacology Faculty of Pharmacy University of Erzincan Binali Yıldırım Erzincan Türkiye

**Keywords:** lactating sheep, milk, pharmacokinetics, tolfenamic acid, withdrawal time

## Abstract

**Objective:**

This study aimed to investigate the plasma and milk pharmacokinetics, as well as the withdrawal time (WT) from milk of tolfenamic acid (2 and 4 mg/kg) following intravenous (IV) administration to eight healthy lactating Akkaraman sheep.

**Methods:**

The trial was conducted in two periods in accordance with a crossover pharmacokinetic design. The concentrations of tolfenamic acid in the plasma and milk were determined using high‐pressure liquid chromatography and evaluated using non‐compartmental analysis. The WT of tolfenamic acid in milk was calculated using the WT 1.4 software.

**Results:**

Compared to the 2 mg/kg dose, plasma volume of distribution at steady state (from 0.43 to 0.50 L/kg), terminal elimination half‐life (from 2.41 to 4.14 h) and dose‐normalized area under the plasma concentration–time curve (AUC_0−∞_, from 9.46 to 30.11 h µg/mL) increased, whereas total body clearance (from 0.21 to 0.13 L/h/kg) decreased at the 4 mg/kg dose. The peak milk concentration (*C*_max_) and AUC_0−∞_ values in milk were 0.26 µg/mL and 0.28 h µg/mL, respectively, for 2 mg/kg, and 0.43 µg/mL and 0.55 h µg/mL, respectively, for 4 mg/kg. Although the dose‐normalized *C*_max_ of milk decreased depending on the dose, no difference was observed in dose‐normalized AUC_0−∞_. The AUC_0−∞ milk_/AUC_0−∞ plasma_ ratio was 0.03 for 2 mg/kg and 0.02 for 4 mg/kg. The WT values calculated for milk at dosages of 2 and 4 mg/kg were 3 and 4 h, respectively.

**Conclusions:**

A decrease in plasma elimination and an increase in plasma concentration of tolfenamic acid were observed depending on the dose. Tolfenamic acid lowly passed into sheep's milk at 2 and 4 mg/kg doses. This study may provide valuable information for clinicians’ decision‐making processes.

## Graphical abstract

Plasma and milk pharmacokinetics of tolfenamic acid in lactating sheep.

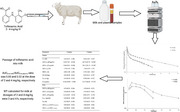

## 1 Introduction

People consume protein‐rich foods, mostly from animal sources (milk, meat and eggs), to meet their nutritional needs (Rana et al. 2019). Milk is an animal food that contains many essential nutrients, such as calcium, protein and vitamin D, and is considered an important part of a balanced diet (Food and Agriculture Organization 2013). According to the Turkish Statistical Institute, there are approximately 43 million sheep used for meat, milk, wool and leather production in Türkiye (Türkiye İstatistik Kurumu [TUIK] 2023). Additionally, sheep contribute approximately 5% of our country's milk production (TUIK 2022).

Veterinary medications are extensively utilized to prevent and treat illnesses in food‐producing animals. These drugs may leave residues in animal food products in the form of parent drugs or metabolites (Pratiwi et al. 2023). Residues refer to drugs or their metabolites that have the potential to accumulate in the tissues or edible parts of treated animals (Pratiwi et al. 2023; Rana et al. 2019). These residues can occur due to incorrect or extra‐label drug use and failure to adhere to drug withdrawal periods (Rana et al. 2019). To minimize or prevent the risk of drug residues in animal foods, maximum residue limits (MRL) must be observed (Pratiwi et al. 2023). Non‐steroidal anti‐inflammatory medications (NSAIDs) are a drug class that is frequently employed in livestock due to their anti‐inflammatory, analgesic and antipyretic properties. However, these drugs have adverse effects on the gastrointestinal, renal, hepatic, cardiovascular and haematologic systems (Pietruk, Jedziniak, and Olejnik 2021), and if residue levels in animal food products exceed the MRL, they may cause these side effects in those who consume them.

Tolfenamic acid, an NSAID of the fenamate group, has analgesic, antipyretic, anti‐inflammatory and anti‐endotoxic properties (Ahmed, Sheraz, and Ahmad 2018; Moilanen and Kankaanranta 1994). Tolfenamic acid shows its pharmacological effect by suppressing the activity of cyclooxygenase (COX) enzymes, which are responsible for the production of prostaglandin (PG) from arachidonic acid (Corum et al. 2018). Tolfenamic acid is used in mastitis, arthritis, omphalectomy, respiratory tract infections, metritis–mastitis agalaxy infections and postoperative analgesia in animals (Committee for Veterinary Medicinal Products 1997; Turk, Tekeli, and Durna Corum 2021a). It is also used in doses of 2 and 4 mg/kg in cases, such as mastitis, bacterial respiratory disease and pathological fever, pain and swelling in sheep (Anonymous 2024a). The MRL value of tolfenamic acid for milk is unknown in sheep but is stated as 50 µg/kg in cattle (The European Commission 2010).

Physiological status due to lactation in females may alter the pharmacokinetics of drugs. However, this change varies depending on the period of lactation and the drug (Martinez and Modric 2010; Kim et al. 2020). Determining the change in the pharmacokinetics of the drug is important in terms of both effectiveness and toxic effects. It has been observed that the pharmacokinetics of tolfenamic acid vary depending on the dose in non‐lactating sheep (Corum et al. 2018). Although tolfenamic acid has been approved for use in lactation sheep (Anonymous 2024a), there are currently no studies providing information on dose‐related plasma and milk pharmacokinetics. The objective of this investigation was to ascertain the plasma and milk pharmacokinetics and withdrawal time (WT) from milk of tolfenamic acid after intravenous (IV) administration to lactating sheep at doses of 2 and 4 mg/kg.

## 2 Materials and Methods

### 2.1 Chemicals

The tolfenamic acid analytical standard, with a purity of at least 98%, was acquired from Sigma‐Aldrich (St. Louis, Mo., USA). Orthophosphoric acid was supplied from Merck (Darmstadt, Germany). Methanol and acetonitrile suitable for high‐performance liquid chromatography (HPLC) were obtained from VWR (Fontenay‐Sous‐Bois, France). For drug administration to sheep, the parenteral formulation (Tolfine 40 mg/mL, Novakim, Kocaeli, Türkiye) was utilized.

### 2.2 Animals

Eight lactating Akkaraman sheep (2–3 years old, 56 ± 4 kg, milk production 330 ± 20 g/day) were utilized. The study started when the animals were on the 45 days of lactation. The sheep were evaluated as healthy by a complete blood count and a general physical examination. Udder health was determined by udder palpation and the California mastitis test. None of the animals selected for the study were administered any medication during the last 30 days. The sheep were placed in pens 7 days prior to the experimental investigation for the acclimatization period. The animals were fed commercial feed in the morning and evening, and water and hay were available ad libitum.

### 2.3 Experimental Design

The trial was conducted in two periods using a cross‐pharmacokinetic design. A 15‐day drug washout period was allowed between periods. Eight sheep were randomly divided into two subgroups. In the first period, 2 mg/kg was administered to one group (*n* = 4), and 4 mg/kg was administered to the other group (*n* = 4). In the second period, the groups were changed to receive different doses, and at the end of the study, eight sheep received both doses. Both doses of tolfenamic acid were administered intravenously (right jugular vein). Blood samples (2 mL) were obtained (left jugular vein) into tubes containing lithium heparin at 0 (control), 0.08, 0.17, 0.25, 0.5, 0.75, 1, 1.5, 2, 3, 4, 5, 6, 8, 10, 12, 18, 24 and 48 h. Milk samples (2 mL) were taken at the times specified above for blood samples. The udder was completely empty at each sampling time. To obtain plasma samples, blood samples were centrifuged (4,000 × *g* for 10 min), and plasma and milk samples were stored at −80°C until analysis of tolfenamic acid.

### 2.4 Tolfenamic Acid Analysis From Plasma and Milk Samples

Tolfenamic acid analysis from plasma and milk samples was conducted using HPLC–ultraviolet (UV) methods previously reported (Corum et al. 2018, 2019) with minor modifications. Shortly, 0.3 mL of acetonitrile was added to microcentrifuge tubes containing 0.2 mL of plasma and milk samples. The microcentrifuge tube was vortexed (60 s) and then centrifuged for 12 min at 12,000 *g*. Following the transfer of the supernatant to autosampler vials, 20 µL were injected into the HPLC. The HPLC system (Shimadzu/Japan) was equipped with a SPD‐20A UV–VIS detector, a CTO‐10A column oven, a DGU‐20A degasser, a SIL‐20A autosampler and a LC‐20AT pump. The HPLC separation of tolfenamic acid was carried out with an inertsil ODS‐3 column (4.6 × 250 mm^2^; 5 µm; GL Sciences, Japan) and maintained at 40°C. The wavelength was set to 289 nm. The mobile phase consisted of acetonitrile (65%) and 0.1% orthophosphoric acid solution (35%), using the isocratic method with a flow rate of 1 mL/min.

Tolfenamic acid calibration standards were linear (*R* ^2^ > 0.9991) for plasma (0.04–40 µg/mL) and milk (0.04–4 µg/mL). To ascertain precision, accuracy and recovery, plasma (0.2, 2 and 20 µg/mL) and milk (0.1, 0.4 and 1 µg/mL) quality control samples of tolfenamic acid were evaluated in six replicates over a span of 6 days. The recovery for plasma and milk was 92% and 87%, respectively. The lower limit of quantification of tolfenamic acid in sheep plasma and milk was 0.04 µg/mL. The coefficients of variance for intra‐day and inter‐day for plasma and milk were ≤7.40% and ≤8.40%, respectively. The intra‐day bias had a range of ±8.0%, whereas the inter‐day bias had a range of ±9.2%.

### 2.5 Pharmacokinetic Analysis

Pharmacokinetic analysis was analysed on plasma and milk tolfenamic acid concentrations using non‐compartmental analysis with WinNonlin 6.1.0.173 software. A non‐compartmental model was used to determine the total body clearance (Cl_T_), volume of distribution at steady state (*V*_dss_), terminal elimination half‐life (*t*_1/2_ *_ʎz_*), mean residence time (MRT), area under the plasma concentration–time curve (AUC) and AUC extrapolated from *t*_last_ to ∞ in % of the total AUC (AUC_extrap_%). The peak milk concentration (*C*_max_), plasma concentration at time 0.08 h (*C*_0_) and time to reach peak concentration (*T*_max_) were calculated directly from the data. The penetration of tolfenamic acid into milk was determined by the AUC_0−∞ milk_/AUC_0−∞ plasma_ ratio.

### 2.6 WT Estimation

The WT of tolfenamic acid in milk was calculated using the WT 1.4 programme created by EMA. The WT was determined using linear regression analysis, considering the reported MRL (50 µg/kg) as the cut‐off, and expressed in hours. The calculation of WT involved the use of a tolerance limit derived from the 95th percentile, with a confidence level of 95%. The WT 1.4 application is designed for evaluations of datasets containing up to seven time points. Tolfenamic acid was detected in milk at 2 mg/kg dose at seven sampling times (0.08–1.5 h) and at 4 mg/kg dose at nine (0.08–3 h) sampling times. Therefore, in the 4 mg/kg dose group, 0.17 and 0.75 h were not included in the analysis. The calculated WT, rounded to the next hour, is indicated among parentheses if the estimated WT was a fraction of an hour.

### 2.7 Statistical Analysis

The *T*_max_ of tolfenamic acid in milk is presented as the median (min–max). The geometric mean (min–max) was used to illustrate other pharmacokinetic data. Prior to statistical analysis, *C*_max_ and AUC_0−∞_ values in the 4 mg/kg group were normalized to 2 mg/kg. The normality and homogeneity of the data were evaluated with the Shapiro–Wilk and Levene tests. The Wilcoxon's rank sum test (SPSS 22.0 programme, IBM Corp., Armonk, NY) was employed to analyse the data. Statistics were considered significant at *p* < 0.05.

## 3 Results

### 3.1 Plasma Pharmacokinetic Parameters

**FIGURE 1:**
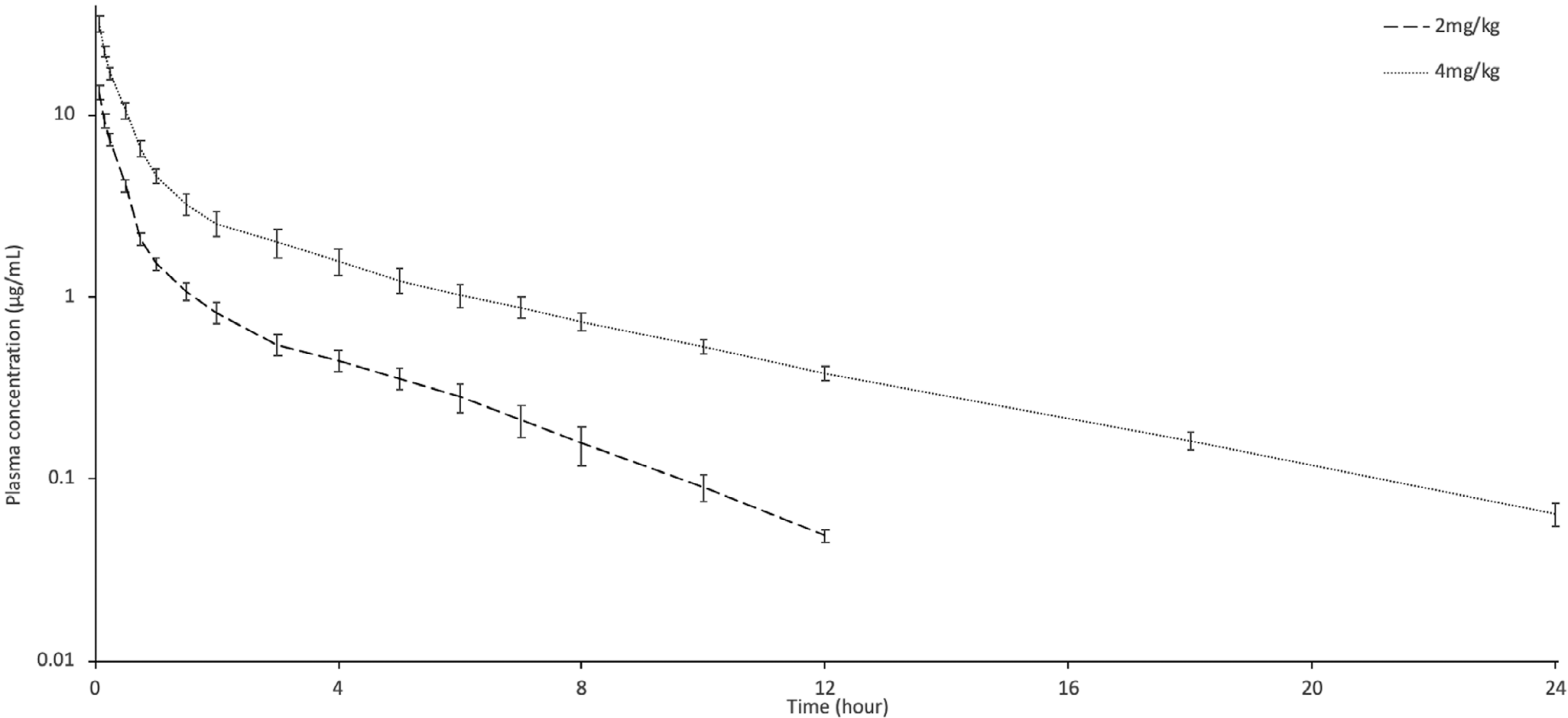
Semi‐logarithmic plasma concentration–time curves of tolfenamic acid following intravenous administrations at doses of 2 and 4 mg/kg to lactating sheep (*n* = 8, mean ± SD).

The semi‐logarithmic plasma concentration–time curves of tolfenamic acid in lactating sheep are shown in Figure 1. Tolfenamic acid was detected in plasma for up to 12 h at a dose of 2 mg/kg and for up to 24 h at a dose of 4 mg/kg. The *C*_0_ was 13.30 µg/mL for 2 mg/kg and 31.77 µg/mL for 4 mg/kg. Plasma concentrations of tolfenamic acid dropped to 0.05 and 0.06 µg/mL at the final sampling times at the doses of 2 and 4 mg/kg, respectively. The pharmacokinetic parameters of tolfenamic acid in lactating sheep are presented in Table 1. The *t*_1/2_ *_ʎz_*, MRT_0−∞_, *V*_dss_, Cl_T_ and AUC_0−∞_ values were 2.41 h, 2.04 h, 0.43 L/kg, 0.21 L/h/kg and 9.46 h µg/mL, respectively, at a dose of 2 mg/kg. At a dose of 4 mg/kg, *t*_1/2_ *_ʎz_*, MRT_0−∞_, *V*_dss_ and AUC_0−∞_ increased, whereas Cl_T_ decreased.

**TABLE 1:** Plasma and milk pharmacokinetic parameters after intravenous administration of tolfenamic acid at doses of 2 and 4 mg/kg to lactating sheep (*n* = 8).

Parameters	2 mg/kg	4 mg/kg
Plasma		
*t*_1/2_ *_ʎz_* (h)	2.41 (2.31–2.50)	4.14 (3.85–4.37)^*^
AUC_0−last_ (h µg/mL)	9.29 (7.83–10.10)	26.73 (25.76–33.09)^*^
AUC_0−∞_ (h µg/mL)	9.46 (8.03–10.30)	30.11 (26.12–33.54)^*^
AUC_extrap_ (%)	1.79 (1.47–2.38)	1.26 (1.07–1.37)
MRT_0−∞_ (h)	2.04 (1.80–2.29)	3.75 (3.66–4.01)^*^
Cl_T_ (L/h/kg)	0.21 (0.19–0.25)	0.13 (0.12–0.15)^*^
*V*_dss_ (L/kg)	0.43 (0.38–0.52)	0.50 (0.44–0.57)^*^
*C*_0_ (µg/mL)	13.30 (10.85–15.08)	31.77 (28.46–37.75)^*^
Milk		
AUC_0−last_ (h µg/mL)	0.25 (0.23–0.26)	0.50 (0.46–0.57)
AUC_0−∞_ (h µg/mL)	0.28 (0.26–0.29)	0.55 (0.52–0.61)
AUC_extrap_ (%)	10.76 (9.95–13.43)	8.95 (6.70–11.97)
*C*_max_ (µg/mL)	0.26 (0.24–0.28)	0.43 (0.38–0.50)^*^
*T*_max_ (h)	0.25 (0.25–0.50)	0.25 (0.25–0.25)
AUC_0−last milk_/AUC_0−last plasma_	0.03 (0.02–0.03)	0.02 (0.02–0.02)
AUC_0−∞ milk_/AUC_0−∞ plasma_	0.03 (0.02–0.04)	0.02 (0.02–0.02)

*Note*: Data were presented as the geometric mean (min‐max) except for *T*_max_, which was presented as the median (min–max).

Abbreviations: AUC, area under the concentration–time curve; AUC_extrap_ %, area under the plasma concentration–time curve extrapolated from t_last_ to ∞ in % of the total AUC; *C*_0_, plasma concentration at time 0.08 h; Cl_T_, total body clearance; *C*_max_, peak plasma concentration; MRT_0−∞_, mean residence time; *t*_1/2_ *_λz_*, terminal elimination half‐life; *T*_max_, time to reach peak plasma concentration; *V*_dss_, volume of distribution at steady state.

* Significantly different from 2 mg/kg dose (*p* < 0.05).

### 3.2 Milk Pharmacokinetic Parameters

**FIGURE 2:**
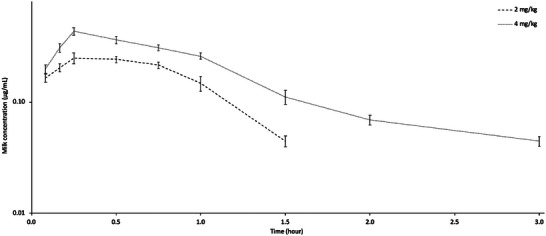
Semi‐logarithmic milk concentration–time curves of tolfenamic acid following intravenous administrations at doses of 2 and 4 mg/kg to lactating sheep (*n* = 8, mean ± SD).

The semi‐logarithmic milk concentration–time curves and pharmacokinetic parameters of tolfenamic acid in lactating sheep are presented in Figure 2 and Table 1, respectively. Tolfenamic acid was detected in milk for up to 1.5 h at a dose of 2 mg/kg and for up to 3 h at a dose of 4 mg/kg, and the concentration at these times was 0.04 µg/mL for both doses. The *C*_max_ and AUC_0−∞_ values in milk were 0.26 µg/mL and 0.28 h µg/mL, respectively, for 2 mg/kg and 0.43 µg/mL and 0.55 h µg/mL, respectively, for 4 mg/kg. Although dose‐normalized *C*_max_ decreased at the 4 mg/kg dose, no difference was seen in dose‐normalized AUC_0−∞_. The AUC_0−∞ milk_/AUC_0−∞ plasma_ ratio was 0.03 and 0.02 at the doses of 2 and 4 mg/kg, respectively.

### 3.3 WT Estimation

**FIGURE 3:**
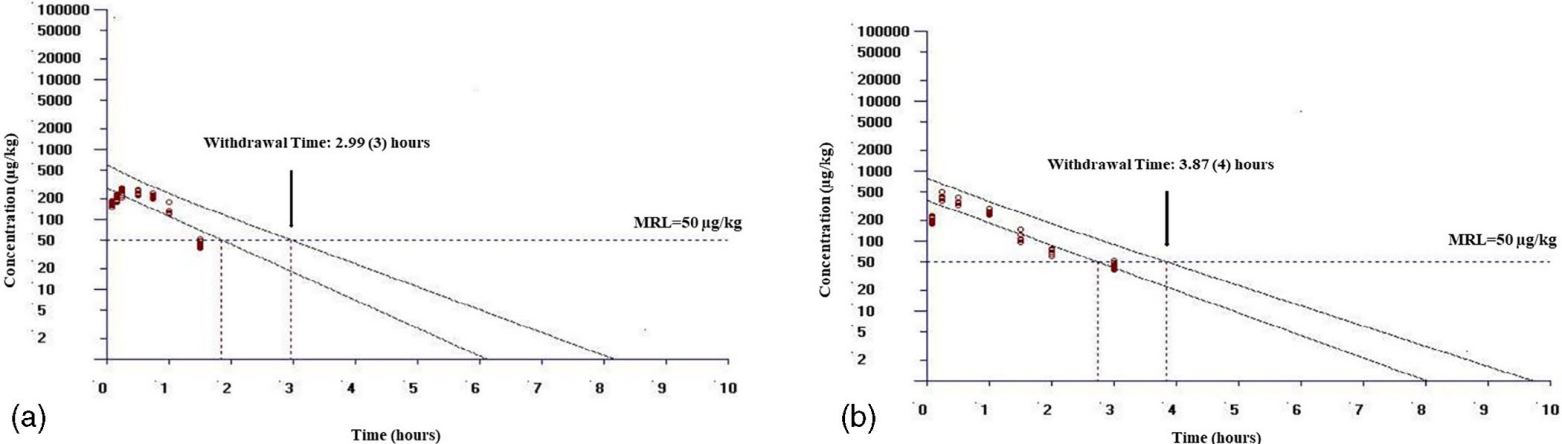
Estimated withdrawal time of tolfenamic acid for milk following intravenous administrations at doses of 2 (a) and 4 (b) mg/kg to lactating sheep.

The WT values for milk obtained from lactating sheep after IV administration of tolfenamic acid are illustrated in Figure 3a,b, respectively. The WT values calculated for milk at doses of 2 and 4 mg/kg were 2.99 (3) and 3.87 (4) h, respectively.

## 4 Discussion

Pharmacokinetic studies conducted in food animals are very important, both in terms of clinical applications and in determining drug residue problems that may endanger public health. The plasma pharmacokinetics of tolfenamic acid has been observed in farm animals, such as sheep, goats and calves, and in poultry, such as partridges, geese and quails (Cetin et al. 2022; Corum et al. 2018; Landoni, Cunningham, and Lees 1996; Tekeli et al. 2020; Turk et al. 2021b, 2021c). However, there is no information about the pharmacokinetics of tolfenamic acid in milk. In this research, the milk and plasma pharmacokinetics of tolfenamic acid and its WT in milk in lactating sheep were revealed for the first time. Although significant differences were determined in plasma pharmacokinetics of tolfenamic acid to lactating sheep at doses of 2 and 4 mg/kg, milk pharmacokinetics and WTs were found to be similar.

Tolfenamic acid is used in inflammatory and painful conditions in lactating sheep. Tolfenamic acid is recommended to be used intravenously and intramuscularly at a dose of 2 mg/kg, whereas it is recommended to be used IV at a dose of 4 mg/kg (Anonymous 2024a). Therefore, we preferred the IV route in this study. No adverse effects were observed after IV administration of tolfenamic acid at doses of 2 and 4 mg/kg in lactating sheep. It has been reported that these doses do not cause any adverse effects in non‐lactating sheep (Yildiz et al. 2019), goats (Tekeli et al. 2020) and calves (Lees et al. 1998).

After IV administration of tolfenamic acid to lactating sheep at a dose of 2 mg/kg, *t*_1/2_ *_ʎz_*, MRT_0−∞_, *V*_dss_ and Cl_T_ were 2.41 h, 2.04 h, 0.43 L/kg and 0.21 L/h/kg, respectively. The *t*_1/2_ *_ʎz_* (1.97 h), MRT_0−∞_ (1.59 h), *V*_dss_ (0.41 L/kg) and Cl_T_ (0.26 L/h/kg) were close to those reported in non‐lactating sheep at the same dose (Corum et al. 2018). These results indicate that lactation has no significant effect on the pharmacokinetics of tolfenamic acid.

Compared to the 2 mg/kg dose, plasma *t*_1/2_ *_ʎz_* (from 2.41 to 4.14 h), *V*_dss_ (from 0.43 to 0.50 L/kg) and dose‐normalized AUC_0−∞_ (from 9.46 to 30.11 h µg/mL) increased and Cl_T_ (from 0.21 to 0.13 L/h/kg) decreased at the 4 mg/kg dose. Similar dose‐dependent alterations have been found in non‐lactating sheep (Corum et al. 2018) and goats (Tekeli et al. 2020). It has been stated that the changes in these pharmacokinetic parameters depending on the dose of tolfenamic acid may be attributed to the change in the binding ratio to plasma proteins, saturation of its metabolism and decrease in clearance due to the change in glomerular filtration (Corum et al. 2018, 2019; Tekeli et al. 2020).

The AUC_milk_/AUC_plasma_ ratio is commonly used to describe the extent to which drugs penetrate into milk. If this ratio is greater than 1, it indicates that the drug accumulates in milk (Verstegen and Ito 2019). The AUC_0−∞ milk_/AUC_0−∞ plasma_ ratio of tolfenamic acid in lactating sheep was 0.03 and 0.02 at the doses of 2 and 4 mg/kg, respectively. This shows that tolfenamic acid passes into sheep milk at very low levels. After IV administration of tolfenamic acid to mice at a dose of 4 mg/kg, the milk/plasma concentration ratio at 0.5 h was 0.24–0.39 (Blanco‐Paniagua et al. 2021). In our study, the milk/plasma concentration at the same time was 0.06 for the 2 mg/kg dose and 0.03 for the 4 mg/kg dose. It is thought that the different ratios of tolfenamic acid passing into milk in sheep and mice may be due to the differences in milk components and amounts between species (Görs et al. 2009; Merlin Junior et al. 2015) and the difference in the lactation period (Martinez and Modric 2010).

Drug excretion into milk occurs through passive diffusion for all drugs and by carrier‐mediated transport mechanisms for some drugs (Verstegen and Ito 2019). Lipophilicity, ionization, protein‐binding affinity and molecular weight are important determinants of drug penetration into milk by passive diffusion (Lee 2007). Tolfenamic acid is an acidic drug (p*K*_a_: 3.7–4.3, Ahmed 2018) and is highly bound to plasma proteins (Corum et al. 2018). Because milk pH (6.6–6.8, Merlin Junior et al. 2015) is lower than blood pH (7.42, Jelinek, Gajdůšek, and Illek 1996), the passage of acidic drugs into milk is generally low. The low ratio of tolfenamic acid passing into milk may be due to its presence in the blood in an ionized state and its high binding to plasma proteins. Similarly, it has been stated that milk/plasma concentrations of NSAIDs vary between 0.006 and 0.37 (Ostensen 1996).

Breast cancer resistance protein (BCRP) is an ATP‐binding cassette efflux transporter that plays a crucial role in excreting substrate medicines from the cell and into milk. BCRP is located on the apical membrane of mammary gland alveolar epithelial cells and is one of the main factors involved in the active secretion of xenotoxins into milk (García‐Lino et al. 2019). Tolfenamic acid is a BCRP substrate, and its distribution in tissues changes depending on the activity of BCRP (Blanco‐Paniagua et al. 2021). However, BCRP does not play a role in the passage of tolfenamic acid into milk (Blanco‐Paniagua et al. 2021). This situation was quite surprising. Tolfenamic acid does not pass into the mammary gland alveolar cells in sufficient concentration due to its high binding to plasma proteins and its presence in ionized form in the blood (Blanco‐Paniagua et al. 2021). Therefore, BCRP may not have a significant contribution to the passage of tolfenamic acid into milk due to the low level of tolfenamic acid in alveolar cells.

Due to the inappropriate use of veterinary medications in food animals, drug residues may be present in foods intended for human consumption. Although the penetration of tolfenamic acid into milk is very low, it has been detected positively with a biochip kit in the milk of mother (Ergen and Yalcın 2019; Yalcin, Gunes, and Yalcin 2020). Because NSAIDs such as tolfenamic acid have adverse effects on the gastrointestinal and cardiovascular systems, kidney and liver, the amount of residues in milk above the MRL value may cause these adverse effects (Pietruk, Jedziniak, and Olejnik 2021). Therefore, the residue of tolfenamic acid in milk should be carefully evaluated. As mentioned earlier, the MRL value of tolfenamic acid for sheep milk is not available. The MRL value for bovine milk is reported as 50 µg/kg (The European Commission 2010). After IV administration of 2 and 4 mg/kg to lactating sheep, milk concentrations of tolfenamic acid were below the MRL value at 1.5 and 3 h, respectively. The WT values in milk at doses of 2 and 4 mg/kg of tolfenamic acid were 3 and 4 h, respectively. It was determined that the WT period in milk was prolonged at the 4 mg/kg dose compared to the 2 mg/kg dose in lactating sheep. However, considering that sheep are milked once or twice a day, it is sufficient to wait one milking period for every two doses of WT. It has been stated that 24 h of WT is sufficient after IV administration of tolfenamic acid in cows (Anonymous 2024b).

## 5 Conclusion

Tolfenamic acid was detected in milk for up to 1.5 h at a dose of 2 mg/kg and for up to 3 h at a dose of 4 mg/kg. Tolfenamic acid lowly passed into sheep's milk at 2 and 4 mg/kg doses. The AUC_0−∞ milk_/AUC_0−∞ plasma_ ratio was quite low at 0.02–0.03. The milk withdrawal period of tolfenamic acid in sheep was 3 and 4 h, respectively, for IV doses of 2 and 4 mg/kg. Therefore, one milking period is sufficient for safe milk consumption in lactating sheep without the risk of residue. However, there is a need to examine the effect of tolfenamic acid in the treatment of mastitis in detail due to its low ratio of excretion into milk after systemic administration in lactating sheep.

## Author Contributions

**Orhan Corum, Kamil Uney**, and **Duygu Durna Corum**: conceptualization, investigation, methodology, resources, supervision, writing—original draft, writing—review and editing. **Devran Coskun** and **Gul Cetin**: investigation; methodology. **Muammer Elmas**: project administration, writing—original draft, writing—review and editing.

## Ethics Statement

The study protocol was approved (2020/62) by the Ethics Committee of the Faculty of Veterinary Medicine (University of Selcuk, Konya, Türkiye) and was conducted in compliance with the European Directive (2010/63/EU).

## Conflicts of Interest

The authors declare no conflicts of interest.

### Peer Review

The peer review history for this article is available at https://publons.com/publon/10.1002/vms3.70047.

## Data Availability

The data that support the findings of this study are available from the corresponding author upon reasonable request.
